# Recompensation of Liver Cirrhosis: Definition, Mechanism, Etiological Differences, Prognosis, and Challenges

**DOI:** 10.1155/cjgh/8512168

**Published:** 2026-03-01

**Authors:** Dan Zhou, Kunyi Ba, Tingting Jia, Yi Shen, Li Yang

**Affiliations:** ^1^ Department of Gastroenterology and Hepatology, and Laboratory of Gastrointestinal Cancer and Liver Disease, West China Hospital, Sichuan University, Chengdu, China, scu.edu.cn

**Keywords:** decompensation, liver cirrhosis, recompensation

## Abstract

The progression from compensated to decompensated cirrhosis has historically been considered irreversible. However, accumulating clinical evidence has given rise to the concept of “recompensation,” which posits that a subset of patients with decompensated cirrhosis may regain a compensated state following successful etiological control, leading to a markedly improved prognosis. This review synthesizes current evidence on cirrhosis recompensation, examining its definition, mechanisms, and etiological specificities, while also addressing the prognosis and persistent challenges in this patient population. Further research is needed to refine the definition of recompensation and elucidate the underlying mechanisms and determinants.

## 1. Introduction

Liver cirrhosis is a chronic pathological condition characterized by diffuse hepatic fibrosis and nodular regeneration, which results from persistent liver injury due to various etiologies such as chronic hepatitis virus infection, metabolic dysfunction–associated steatotic liver disease (MASLD), and alcoholic liver disease (ALD), with many patients having multiple overlapping etiologies [1]. Clinically, liver cirrhosis progresses through two distinct phases: a compensated phase, which is often asymptomatic, and a decompensated phase, marked by the development of complications such as hepatic encephalopathy (HE), esophageal and gastric variceal bleeding (EGVB), and ascites [2]. Decompensation arises from the combined effects of architectural distortion, which increases intrahepatic vascular resistance and portal pressure, and progressive hepatic dysfunction [3]. Cirrhosis poses a substantial global health burden and is a leading cause of mortality worldwide [4]. Critically, the transition from compensation to decompensation signifies a pivotal worsening of prognosis, with median survival decreasing from 10 to 12 years to approximately 2–4 years, accompanied by a severely impaired quality of life [5].

Previously considered irreversible, the course of decompensated cirrhosis has shown signs of reversibility not only in selected patients awaiting liver transplantation (LT)—leading to delisting—but also in a broader decompensated cirrhotic population following control of the underlying etiology. A substantial proportion of these patients maintain sustained clinical and biochemical improvement without further decompensation events, a phenomenon now termed “recompensation” [6–8]. This review aims to synthesize current knowledge on cirrhosis recompensation, examining its definition, potential mechanisms, predictive factors, and prognosis across different etiologies, and to discuss the ongoing challenges in this evolving field.

## 2. Definition of Recompensation in Cirrhosis

**FIGURE 1:**
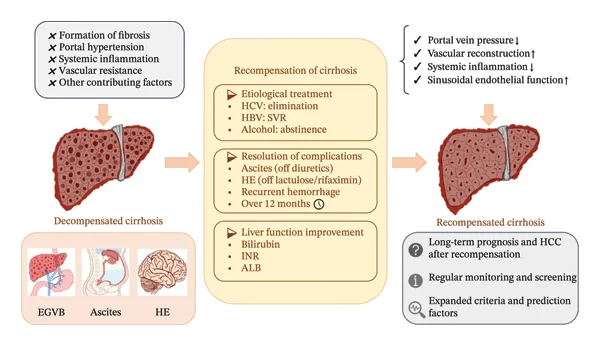
An overall profile for recompensation defined by Baveno VII criteria and potential mechanisms. Abbreviations: ALB, albumin; EGVB, esophageal and gastric variceal bleeding; HBV, hepatitis B virus; HCC, hepatocellular carcinoma; HCV, hepatitis C virus; HE, hepatic encephalopathy; INR, International Normalized Ratio.

The concept of recompensation, defined as at least partial restoration of liver structure and function, has been formally incorporated into recent national and international guidelines and consensus statements [9–13]. The 2021 Baveno VII consensus provides the most specific and widely referenced criteria for the recompensation of cirrhosis (Figure 1), stipulating that all the following requirements must be met: (i) removal or suppression of the primary etiology, such as viral suppression/elimination and alcohol abstinence; (ii) resolution of major complications (variceal bleeding, HE, and ascites) for at least 12 months off therapy; (iii) sustained improvement in liver function (measured by serum bilirubin, International Normalized Ratio (INR), and albumin (ALB)) [9]. To specify precise biochemical thresholds, Wang et al. conducted a 120‐week follow‐up study in patients with hepatitis B–related decompensated cirrhosis and suggested that liver function tests meeting the biochemical range of Child‒Pugh A (total bilirubin < 34 μmol/L, INR < 1.5, and ALB > 35 g/L) and/or a Model for End‐Stage Liver Disease (MELD) score < 10 could serve as practical standards for defining stable liver function improvement [14]. However, while these specific thresholds are logically derived from the well‐established Child–Pugh classification, they await formal validation in large, independent cohorts.

Although the Baveno VII criteria are increasingly adopted, some studies employ modified definitions tailored to their specific research contexts [15–17]. A unifying theme across all criteria is the imperative to control the primary etiology and the expectation of sustained clinical and biochemical stability as the hallmark of successful recompensation.

## 3. Pathophysiological Mechanisms

The pathogenesis of liver cirrhosis is centered on hepatic stellate cell activation, as a key driver of extracellular matrix secretion and increased intrahepatic sinusoidal pressure. This process is compounded by the degradation of normal hepatic extracellular matrix, excessive accumulation of fibrous tissue, vascular remodeling, and the action of various profibrogenic cytokines [1]. A foundational concept is that liver fibrosis can regress following the removal of the underlying injurious stimulus [18, 19]. Bedossa suggested that the reversal of liver cirrhosis is histologically characterized by collagen degradation, hepatocyte regeneration, and the reconstruction of lobular structure [20]. Providing a crucial histopathological blueprint for this reversal, the seminal work by Wanless et al. described a “hepatic repair complex” in explanted livers [21]. This complex includes features such as perforated delicate septa, indicating ongoing resorption of fibrous scaffolds; hepatic vein remnants with prolapsed hepatocytes and minute regenerative nodules, demonstrating hepatocyte repopulation; and portal tract remnants, reflecting the remodeling of vascular structures. Collectively, these observations illustrate a slow but continuous process of structural reversal upon cessation of injury, which forms the pathological basis for the functional recovery that defines clinical recompensation.

### 3.1. Decreased Portal Vein Pressure and Recovered Liver Sinusoidal Endothelial Function

Portal hypertension, a key driver of decompensation, results from increased intrahepatic vascular resistance due to structural changes (fibrosis, vascular remodeling) and functional disturbances (endothelial dysfunction), which in turn promote the formation of portosystemic collateral circulation and shunts [1, 22]. A hepatic venous pressure gradient (HVPG) ≥ 10 mmHg is closely associated with decompensating events, while reducing it below 12 mmHg significantly lowers the risk of such complications [9]. Etiological control—such as antiviral therapy or alcohol abstinence—serves as a foundational strategy to achieve this reduction. By durably suppressing hepatic inflammation and promoting fibrosis regression, these interventions effectively reduce intrahepatic resistance and consequently lower HVPG, as demonstrated in patients with hepatitis B, hepatitis C, and alcohol‐associated cirrhosis [23–26]. Moreover, the effective control of portal hypertension may help restore gastrointestinal mucosal barrier integrity, thereby reducing bacterial translocation and subsequent systemic inflammation. This mitigation of extrahepatic complications further contributes to the promotion of hepatic recompensation [27].

### 3.2. Control of Systemic Inflammation

Systemic inflammation is a well‐established driver of disease progression in cirrhosis, while direct evidence linking its resolution to recompensation remains limited and is an area of active investigation [28]. Markers such as IL‐6 are significantly elevated in decompensated advanced chronic liver disease [29]. Notably, Tonon et al. revealed that levels of IL‐6, IL‐1β, and IL‐10 in recompensated patients were significantly lower than those in the decompensated stage and were comparable to levels observed in compensated patients [30]. This suggests that successful etiological control is associated with a reversal of the systemic proinflammatory state. Conversely, the persistence of inflammation appears to predict adverse outcomes even after clinical improvement. Monteiro et al. found that recompensated patients with detectable levels of inflammasome‐derived cytokines (IL‐1α and IL‐1β) retained a higher risk of subsequent acute‐on‐chronic liver failure (ACLF) [31]. Collectively, although the evidence base is still evolving, these findings position the resolution of systemic inflammation as a plausible and important mechanistic component linking etiology control to stable recompensation.

### 3.3. Characteristic Expression of Regeneration‐Related microRNAs (miRNAs)

Among patients with hepatitis C who achieved a sustained virologic response (SVR) with direct‐acting antiviral agent (DAA) treatment and subsequent improvement in Child‒Pugh scores, a signature of downregulation of miRNA‐26a and miRNA‐200b alongside upregulated miRNA‐126 was observed compared to those without clinical improvement. These specific miRNA changes are associated with decreased apoptosis and promoted cell proliferation and angiogenesis. Notably, these miRNA signatures mirror patterns observed in patients who spontaneously recovered from paracetamol‐induced acute liver failure, suggesting potential common regenerative mechanisms [32].

## 4. Achievement of Recompensation in Cirrhosis With Different Etiologies

Research on recompensation in decompensated cirrhotic patients has primarily focused on etiologies with well‐established therapeutic targets, including viral hepatitis (hepatitis B virus (HBV), hepatitis C virus (HCV)), and ALD. For these conditions, recompensation criteria have been validated or proposed based on consistent clinical improvement observed following etiology control (Table 1). In contrast, evidence for recompensation remains limited for other causes of cirrhosis, such as MASLD, autoimmune liver diseases, genetic/metabolic disorders (e.g., Wilson’s disease), and drug‐induced or parasitic liver injury. The development of standardized recompensation criteria for these etiologies is challenged by heterogeneous disease mechanisms and the frequent lack of targeted, curative therapies.

**TABLE 1:** Summary of current evidence evaluating recompensation of cirrhosis.

Author/year/country/design	Patients and etiology	Definition of recompensation	Incidence of recompensation	Factors associated with recompensation	Outcomes of recompensated patients
Aravinthan et al. [7]/2017/Canada/Retrospective	Transplant waitlisted Patients (ALD, HCV, MASLD, HBV, AIH, Others) *n* = 935	• Absence of ascites/hepatic hydrothorax/peripheral edema (off diuretics) • Absence of HE (off prophylactic treatment) • An improvement in the MELD score to < 15 • Hold for at least 6 months	Overall delisting: 77/935 (8.2%) ALD: 16.5% HBV: 5.5% HCV: 5.0% MASLD: 2.6%	(In ALD): Female sex, bilirubin, INR, creatinine, serum sodium, MELD (< 20), ALB (≥ 32 g/L), PLT	4 recompensated patients were again recommended for LT
Macken et al. [33]/2019/UK/Prospective	Decompensated hepatitis C cirrhosis *n* = 39	• Clinical improvement and no decompensation during follow‐up	20/39 (51.3%)	Serum creatine at baseline	No data provided
Xu et al. [34]/2021/China/Prospective	Recompensated patients (HBV, HCV, ALD, AIH, Others) *n* = 553 Acute decompensated patients *n* = 3400	• Clinically stable state for at least 1 year • No recurrent decompensation events (previously treated/controlled ascites)	No data provided	ALB (40 g/L), total protein, hemoglobin, basophil percentage, ALT, NLR, diabetes	No data provided
Wang et al. [14]/2022/China/Prospective	Decompensated hepatitis B cirrhosis *n* = 283	• Baveno VII criteria for recompensation • MELD < 10 and/or Child‒Pugh A: ‐ ALB > 35 g/L ‐ INR < 1.5 ‐ Bilirubin < 34 μmol/L	Baveno VII criteria: 171/283 (60.4%) Additional MELD < 10/Child‒Pugh A: 159/283 (56.2%)	AST, sodium, PLT, and ALB at 48 weeks of therapy	31 (91.2%) remained compensated, and 3 (8.8%) were diagnosed with HCC among 34 recompensated patients followed up to 144 weeks
Kim et al. [35]/2022/Korea/Retrospective	Decompensated hepatitis B cirrhosis *n* = 311 ‐ Derivation cohort *n* = 152 ‐ Validation cohort *n* = 159	Recovery to a Child‒Pugh score of 5 for at least 2 months	193/311 (62.1%)	Bilirubin, no severe complications, AFP, ALT, INR, duration of decompensation	No data provided
He et al. [36]/2023/China/Retrospective	Hepatitis B cirrhosis with the first decompensated event of ascites or variceal bleeding *n* = 130	No second/further decompensation during follow‐up	Immediate‐treatment group: 39.0% On‐treatment group: 9.8% Delayed/no treatment group: 6.6%	Two years free of complications	Of patients free of 2‐year decompensated complications: • 71.2% maintained stable recompensation • Cumulative incidence of death/LT and HCC: 2.9% and 12.6%
Li et al. [37]/2023/China/Retrospective	First decompensated hepatitis B cirrhosis *n* = 265	Ascites recompensation: • Negative HBV DNA • Improved liver function • Ascites regression (off diuretics)	59.7%, 70.0%, 52.3%, 59.4%, 46.2% at 12, 24, 36, 48, and 60 months	ALT, HBV DNA	No data provided
He et al. [38]/2023/China/Retrospective	Treatment‐naive HBV‐related decompensated patients *n* = 383 Ascites group *n* = 298 Bleeding group *n* = 85	• Maintaining free of decompensation for 1 year • Achieving Child–Pugh A and/or MELD < 10	Ascites group: 170/298 (57.0%) Bleeding group: 33/85 (38.8%)	No data provided	• Second decompensation, further ascites rate, further bleeding rate at Year 5: 26.7%, 24.0%, and 6.0% in the ascites group vs. 43.3%, 16.0%, 33.9% in the bleeding group. • Death/LT rate at Year 5 in ascites/bleeding group: 0.6% vs. 3.0%
Hofer et al. [39]/2023/Austria/Retrospective	Decompensated alcohol‐associated cirrhosis *n* = 204	Baveno VII criteria for recompensation	37/265 (18.1%)	ALB, MAP, HVPG, Child‒Pugh score, BMI	• Recompensation decreased a 91% risk of liver‐related mortality and a 78% risk of all‐cause mortality. • HCC occurred in 5.4% of recompensated patients vs. 6.6% in decompensated patients
Wen et al. [40]/2023/China/Retrospective	Hepatitis B cirrhosis with ascites for the first decompensation *n* = 351 Training cohort *n* = 279 Validation cohort *n* = 72	• Absence of ascites (off diuretics, > 1 year) • No other decompensated complications/HCC/LT occurred	150 (42.7%)	Age, ALT, ALB, sodium, AFP, SVR	No data provided
Hui et al. [41]/2023/China/Retrospective	Compensated cirrhosis *n* = 3327 (71%) Decompensated cirrhosis *n* = 1109 Recompensated cirrhosis *n* = 265	Baveno VII criteria for recompensation	No data provided	No data provided	• 18 patients died and 1 underwent LT at a median follow‐up of 5 years • 3‐, 5‐year transplant‐free survival: 72.5%; 51.3%
Gao et al. [42]/2023/China/Retrospective	Patients with decompensated cirrhosis who received TIPS for variceal bleeding or refractory ascites (viral, alcohol, other) *n* = 64	• Baveno VII criteria for recompensation • MELD < 10 and/or Child‒Pugh A: ‐ ALB > 35 g/L ‐ INR < 1.5 ‐ Bilirubin < 34 μmol/L	20 (31%)	Age, post‐TIPS portal pressure gradient < 12 mmHg	No data provided
Zhang et al. [43]/2024/China/Retrospective	Hepatitis B cirrhosis *n* = 404 Compensated *n* = 233 (57.7%) Recompensated *n* = 100 (24.8%) Decompensated *n* = 71 (17.6%)	Baveno VII criteria for recompensation	100 (58.5%)	Age, ALT, male sex	HCC development at 2, 4, and 6 years: 1.2%, 5.2%, and 24.5%
Hofer et al. [44]/2024/Austria/Retrospective	PBC with decompensated cirrhosis *n* = 42	• Baveno VII criteria for recompensation • Successful etiological treatment: ‐ Bilirubin normalization ‐ ALP reduction to ≤ 1.5 ULN	All: 7 (16.7%) With UDCA response at 1 year: 5/12 (41.7%) Without UDCA response at 1 year: 2/21 (9.5%)	ALP, bilirubin, MELD‐Na	• 4/7 patients presented with liver‐related complications after developing hepatic malignancy and/or portal vein thrombosis during follow‐up. • 2 patients died at 18 and 29 months after recompensation
Sánchez et al. [45]/2024/Spain/Retrospective	Patients with decompensated cirrhosis undergoing TIPS (alcohol, HCV, MASLD, mixed, other) *n* = 116	Baveno VII criteria for recompensation	At 1 year: 24% (28/116), At 6 months: 27% (31/116)	Liver function (MELD), LDL‐cholesterol, white cell count, PLT	• Risk of HCC at 5 years was lower than that of non‐recompensated patients. • Survival at 5 years was higher than that of non‐recompensated patients. • Similar rates of HCC and survival to compensated patients
Premkumar et al. [46]/2024/Prospective	Decompensated hepatitis C cirrhosis *n* = 1152	Baveno VII criteria for recompensation (after SVR‐12)	284 (24.7%)	Bilirubin, INR, absence of large esophageal or gastric varices	Achieving recompensation reduced risk of deaths
Piecha et al. [47]/2025/Germany/Prospective	Patients with liver cirrhosis who received TIPS for recurrent or refractory ascites (ALD, MASH, cryptogenic, other) *n* = 61	• No standardized criteria • Therapeutic success of TIPS defined as ascites control, and transplant‐free survival at 6 months	48 (79%, ascites control)	Epithelial cell death markers m30 and m65	• No data provided
Zhang et al. [48]/2025/China/Retrospective	HBV‐related acute‐on‐chronic liver failure *n* = 136	• Baveno VII criteria for recompensation • MELD < 10 and/or Child‒Pugh A: ‐ ALB > 35 g/L ‐ INR < 1.5 ‐ Bilirubin < 34 μmol/L	56 (41.2%)	Bilirubin, INR, AFP, age	• 10.5% developed HCC within 5 years • Compared with the non‐recompensation group, the 60‐month survival rate was significantly higher
Sánchez‐Torrijos et al. [49]/2025/Spain/Retrospective	Hepatitis C cirrhosis (achieving SVR) *n* = 216	• Baveno VII criteria for recompensation • Improvement in liver function: MELD score < 10	137 (63.4%)	Child–Pugh score, the number of past decompensating events	• Survival was better in recompensated patients than in decompensated patients, worse than in compensated patients. • HCC occurrence: 14.4%
Arvaniti et al. [50]/2025/Multicenter/Retrospective	Decompensated AIH cirrhosis *n* = 232	• The absence of ascites after diuretic discontinuation and/or the absence of overt HE after resolution of the initial event once prophylactic treatment was withdrawn, and the absence of recurrent variceal bleeding (for at least 12 months)	49%	Treatment response	The LT‐free survival rate was significantly higher than non‐recompensated patients: • At 12 months: 99% vs. 49% • At 36 months: 97% vs. 37%
Tonon et al. [30]/2025/Italy/Retrospective	Decompensated cirrhosis with etiological cure (alcohol, HCV, HBV) *n* = 298	• Baveno VII criteria for recompensation • Expanded criteria: resolution of decompensating events for > 12 months while still on treatment with lactulose/rifaximin and/or a stable low dose of diuretics	Baveno VII criteria: 21 (7%) Expanded criteria: 112 (37.6%)	MELD, BMI, Hemoglobin	• Risk of death similar to compensated cirrhosis
Semmler et al. [51]/2025/Multicenter/Retrospective	Patients with decompensated HCV cirrhosis (SVR) *n* = 361	Baveno VII criteria for recompensation	132 (36.6%)	ALB, diabetes	• Lower risk of liver‐related death and portal vein thrombosis. • The risk of HCC did not decrease
Bai et al. [52]/2025/China/Retrospective	Patients with decompensated cirrhosis who underwent TIPS (HBV, HCV, alcohol, Schistosoma, AIH, others) *n* = 621	Baveno VII criteria for recompensation (no clear stipulation on the mandatory requirement for discontinuation of the medication)	126 (20.3%)	Sarcopenia, PLT	Significantly higher cumulative survival rate compared to non‐recompensated patients
Lin et al. [53]/2025/China/Retrospective	Decompensated PBC cirrhosis *n* = 401	• UDCA treatment response (ALP < 1.67 × ULN after 12 months) • Resolution of decompensating events (off‐related medication for at least 12 months) • Stable improvement in liver function	105 (26.2%)	Lymphocyte‐to‐monocyte ratio	• No data provided
Chen et al. [54]/2025/China/Retrospective	Decompensated AIH cirrhosis accepting *n* = 220	• Complete biochemical response with normalization of transaminases and IgG under IST • Resolution of decompensating events (off‐related medication for at least 12 months) • Liver function improvement (Child–Pugh A)	68 (30.9%)	Ascites as the only complication, IgG, Bilirubin, PLT	• Significantly reduced risk of LT/death, comparable to compensated patients. • 5.9% of recompensated patients experienced further decompensation

*Note:* AFP, alpha‐fetoprotein; AIH, autoimmune hepatitis; ALB, albumin; ALP, alkaline phosphatase; ALT, alanine aminotransferase; AST, aspartate aminotransferase; HCC, hepatocellular carcinoma; IgG, immunoglobulin G; IST, immunosuppressive therapy; LDL, low‐density lipoprotein‐cholesterol; MASH, metabolic dysfunction–associated steatohepatitis; MASLD, metabolic dysfunction–associated steatotic liver disease; MELD, model for end‐stage liver disease; UDCA, ursodeoxycholic acid.

Abbreviations: ALD, alcoholic liver disease; BMI, body mass index; HBV, hepatitis B virus; HCV, hepatitis C virus; HE, hepatic encephalopathy; HVPG, hepatic venous pressure gradient; INR, International Normalized Ratio; LT, liver transplantation; MAP, mean arterial pressure; NLR, neutrophil‐to‐lymphocyte ratio; PBC, primary biliary cholangitis; SVR, sustained virologic response; TIPS, transjugular intrahepatic portosystemic shunt; ULN, upper limit of normal.

### 4.1. Hepatitis B Cirrhosis

Untreated patients with decompensated HBV‐related cirrhosis have a poor prognosis. The advent of potent nucleos(t)ide analogs (NAs), which enable long‐term viral suppression, has revolutionized management and significantly improved outcomes [55–57]. A Korean study of 707 patients with hepatitis B cirrhosis following a first decompensation event demonstrated that antiviral treatment led to a reduction in MELD scores over 60 months of follow‐up, with 33.9% of patients improving sufficiently to be removed from the liver transplant waiting list (WL). Among 295 patients with subsequent long‐term monitoring, LT‐free survival rates at 5 and 10 years were 60.1% and 45.7%, respectively, underscoring the potential for prolonged survival with effective antiviral therapy [58].

Subsequent studies have quantified recompensation rates. In a multicenter study by Wang et al., 60.4% of 283 entecavir‐treated patients with decompensated hepatitis B cirrhosis (with ascites as the initial decompensation) met Baveno VII recompensation criteria by Week 120. Notably, no patient with a MELD score > 15 at Week 48 achieved recompensation within the study period, suggesting the diminished likelihood of recovery in patients with severe liver dysfunction [14]. Similarly, Kim et al. found that 62.1% of decompensated patients achieved a sustained Child–Pugh score of 5 after NA therapies [35]. The potential for reversal is further supported by studies focusing on specific complications. Li et al. demonstrated “ascites recompensation” (resolution of ascites off diuretics alongside improved liver function and undetectable HBV DNA) in a substantial proportion of patients [37]. He et al. compared outcomes based on the initial decompensation event, finding that HBV cirrhotic patients presenting with ascites were more likely to achieve recompensation than those presenting with bleeding (57.0% vs. 38.8%). Furthermore, recompensated patients in the ascites group had a lower 5‐year rate of second decompensation than those in the bleeding group (26.7% vs. 43.3%) [38]. Collectively, these data confirm that antiviral therapy can improve liver function and alleviate clinical complications in patients with decompensated hepatitis B cirrhosis, with a significant subset of patients achieving recompensation.

The concept of recompensation is also being explored in the more severe context of ACLF, predominantly in HBV populations. Yuan et al. analyzed 461 ACLF patients (HBV–ACLF: 49.2%) and found that those who transitioned to a recompensated state (73/461) had milder clinical features and 1‐year survival rates comparable to compensated patients [59]. Similarly, Zhang et al. reported that 41.2% (56/136) of HBV‐related ACLF patients achieved recompensation within 1 year using Baveno VII criteria [48]. These patients had lower bilirubin, elevated alpha‐fetoprotein (AFP), and fewer complications; however, 10.5% developed hepatocellular carcinoma (HCC) during follow‐up, necessitating ongoing surveillance. Mechanistically, Monteiro et al. provided a crucial insight that even in clinically stable, recompensated patients, the presence of detectable IL‐1β—a marker of canonical inflammasome activation—conferred a 1.8‐fold higher risk of subsequent ACLF, suggesting that subclinical inflammation may persist despite overt clinical stability [31]. Therefore, while recompensation is achievable in ACLF patients, the distinct pathophysiological mechanisms triggered by acute injury warrant tailored monitoring strategies for this population.

### 4.2. Hepatitis C Cirrhosis

The advent of DAAs marked a new era in the management of chronic HCV infection, showing significant efficacy even in patients with prior decompensation. Evidence consistently demonstrates that achieving an SVR with DAAs in this population translates into significant clinical benefits, including reduced mortality, improved liver function, and resolution of complications such as ascites, compared to untreated controls or those receiving only symptomatic care [60–63]. A pivotal consequence of this clinical improvement has been the delisting of a considerable number of HCV‐related decompensated patients from the liver transplant WL due to sustained recovery [8, 64].

Subsequent studies have specifically quantified recompensation rates following SVR, though reported figures vary considerably (24.7%–64.7%) due to differences in cohort characteristics and definitions. Early cohort studies in relatively select populations reported high rates of functional recovery, with an Italian cohort study demonstrating 61.8% recompensation rate (Child–Pugh B to A) among HCV‐related cirrhotic patients with Child‒Pugh B who achieved SVR [65]. Real‐world UK data revealed a 51% recompensation rate among 39 patients with baseline decompensated HCV‐related cirrhosis following DAA therapies [33]. In a Spanish multicenter cohort of 216 decompensated patients, Sánchez‐Torrijos et al. observed a 63.4% recompensation rate at 12 months post‐SVR [49]. In contrast, other studies that enrolled more severe populations and applied stricter Baveno VII criteria report more modest rates. The large prospective study by Premkumar et al., which included only patients with Child–Pugh B/C cirrhosis (enriched with the harder‐to‐treat genotype 3), found that only 24.7% achieved recompensation according to Baveno VII criteria (median 16.5 months after SVR‐12). Notably, 19% experienced further decompensation despite virological cure [46]. Similarly, Semmler et al. reported that 28.8% of 361 European patients achieved recompensation over a median 1.9 years, and this proportion increased to 36.6% with extended follow‐up (median 8.4 years) [51]. This wide variability in recompensation rates can be attributed to factors such as higher baseline disease severity, specific HCV genotypes, the stringency of the recompensation criteria applied, and the duration of follow‐up.

### 4.3. Hepatitis D Cirrhosis

Chronic hepatitis D, caused by hepatitis D virus (HDV) superinfection in HBV carriers, is associated with accelerated fibrosis progression and a higher risk of decompensation [66]. Emerging evidence suggests that targeted antiviral therapy can also facilitate clinical improvement in this population. In a case series of 19 patients with decompensated HDV cirrhosis, treatment with the HDV entry inhibitor induced a virologic response in 74% of patients and alanine aminotransferase (ALT) normalization in 74%, while 47% of patients showed Child–Pugh B‐to‐A improvement, alongside ascites resolution in 58% of cases. These clinical improvements are consistent with the principles of recompensation. However, LT was still required in some nonresponders, underscoring the need for early intervention before advanced decompensation occurs. Further studies are needed to establish HDV‐specific recompensation criteria and to evaluate the long‐term sustainability of these clinical benefits [67].

### 4.4. Alcohol‐Associated Cirrhosis

Sustained alcohol abstinence is the cornerstone of treatment for ALD, leading to significant reductions in Child–Pugh score and portal pressure [68]. This potential for recovery is exemplified by the fact that patients with ALD are most likely to be delisted from the WL of LT following clinical improvement. The mandatory 6‐month abstinence period required prior to LT listing inherently provides a window for such recovery to occur [6].

The reported rates of recompensation in alcohol‐associated cirrhosis vary across studies. Aravinthan et al. found that 16.5% of waitlisted ALD patients were delisted due to recompensation. Patients with lower MELD scores and higher serum ALB levels at listing were more likely to be delisted due to recompensation [7]. In contrast, a Spanish study reported an 8.6% delisting rate due to improvement, with lower MELD scores and higher platelet counts being key predictors [6]. A multicenter study by Hsu et al. demonstrated that among severe alcohol‐associated hepatitis patients (median MELD‐Na 31) who were declined for early LT due to psychosocial reasons (56%), only 10% achieved recompensation (defined as MELD < 15 without complications) at 1 year, while among those initially declined due to clinical improvement (21%), the probability was higher (28%) [69]. Hofer et al. demonstrated that among 204 patients with decompensated alcohol‐associated cirrhosis, 18.1% achieved recompensation over an average follow‐up of 24.4 months, which was associated with a > 90% reduction in liver‐related mortality [39]. Collectively, these findings indicate that while abstinence can induce clinical recovery, the rate of recompensation in alcohol‐associated cirrhosis is lower than that achieved in viral hepatitis following etiology control, underscoring the persistent need for LT assessment in this population.

### 4.5. MASLD Cirrhosis

MASLD encompasses a spectrum of liver injury, ranging from simple steatosis to metabolic dysfunction–associated steatohepatitis (MASH) and potentially progressing to advanced fibrosis and cirrhosis [70]. The natural history of MASLD before decompensation is considered bidirectional and reversible [71], with studies confirming that MASH‐related liver fibrosis can regress following weight loss [72]. The most compelling evidence for disease reversal comes from bariatric surgery, a potent metabolic intervention. Lassailly et al. demonstrated in a prospective study of 180 severely obese patients with biopsy‐proven MASH that bariatric surgery induced long‐term resolution of MASH (84%) and regression of fibrosis (70%, with 56% achieving complete fibrosis disappearance) over 5 years, highlighting the potent effect of sustained metabolic intervention. However, the generalizability of these findings to patients with established decompensated cirrhosis is limited, as the study population was predominantly noncirrhotic, and bariatric surgery is often contraindicated in advanced liver disease [73]. Consequently, robust evidence specifically addressing recompensation in decompensated MASLD cirrhosis remains scarce, likely due to the complexity of reversing long‐standing metabolic drivers. Nevertheless, lifestyle adjustments remain the foundational management strategy [74]. Furthermore, patients with MASLD cirrhosis on liver transplant WL may still be delisted owing to clinical improvement [6, 7]. Given the rising global prevalence of MASLD, there is a pressing need for more research to define recompensation rates and influencing factors in individuals with MASLD cirrhosis.

### 4.6. Primary Biliary Cholangitis (PBC) Cirrhosis

The potential for recompensation in PBC has been explored in the context of treatment with ursodeoxycholic acid (UDCA). A recent study by Hofer et al. investigated this in decompensated PBC patients and used bilirubin normalization and alkaline phosphatase (ALP) ≤ 1.5 times the upper limit of normal (ULN) as indicators of successful biochemical response. Ultimately, 16.7% (7/42) of patients achieved recompensation [44]. A larger subsequent study by Lin et al. (*n* = 401) reported a higher recompensation rate of 26% following at least 12 months of UDCA therapy, and a higher lymphocyte‐to‐monocyte ratio was identified to be significantly associated with successful recompensation (*p* < 0.001). Notably, recompensated patients exhibited markedly improved survival compared to non‐recompensated counterparts at 1, 3, and 5 years (96.97%, 92.23%, 86.98% vs. 78.04%, 58.85%, 45.77%, *p* < 0.001) [53]. However, it is crucial to note that UDCA functions as a first‐line disease‐modifying therapy that effectively alters the disease course, rather than a true etiological cure. Consequently, the concept of recompensation in PBC, while demonstrating significant clinical improvement, requires careful interpretation within this therapeutic context.

### 4.7. Autoimmune Hepatitis (AIH) Cirrhosis

Recent studies have investigated the effects of immunosuppressive therapy on recompensation in patients with AIH‐related decompensated cirrhosis. In the study by Chen et al., 30.9% of 220 patients treated with immunosuppressive therapy achieved recompensation over a median follow‐up of 47 months, as defined by modified Baveno VII criteria (sustained resolution of clinical complications for ≥ 12 months and restoration of liver function to Child–Pugh A). Recompensated patients exhibited a significantly reduced risk of LT or death, with survival comparable to compensated patients [54]. In contrast, an international multicenter study by Arvaniti et al. (*n* = 214) reported a higher recompensation rate of 49% (median follow‐up: 27 months). This discrepancy is likely attributable to the less stringent criteria used in the latter study, which required only the absence of clinical complications for at least 12 months without mandating sustained biochemical normalization [50].

## 5. Prediction of Cirrhosis Recompensation

Despite successful etiological control, a significant proportion of decompensated patients failed to achieve recompensation. The early identification of patients unlikely to recompensate is therefore crucial for optimizing management strategies. A range of clinical and biochemical variables have been associated with recompensation likelihood. Serum ALB consistently emerges as a powerful predictor across etiologies. A prospective study highlighted that serum ALB ≥ 34 g/L at Week 24 of antiviral therapy was a key predictor of subsequent recompensation at Week 120 in HBV‐related cirrhosis [75]. Similarly, a multicenter retrospective analysis by Xu et al. determined, via a decision‐tree model with high specificity (86.8%) and sensitivity (92.6%), that ALB was the most influential clinical index for recompensation, identifying a threshold of 40 g/L [34]. A case–control study by Korobka et al. found that a higher serum ALB concentration and white blood cell count at the time of LT listing served as independent predictive markers for subsequent delisting due to recompensation [76]. Beyond ALB, other markers provide prognostic insights through distinct pathways: classic liver function tests (e.g., bilirubin, INR), the achievement of virological responses (e.g., SVR in HCV), and dynamic markers such as elevated AFP (potential indicator of hepatic regeneration) and elevated ALT (which may reflect a vigorous antiviral immune response). Patient‐specific factors, including age, presence of diabetes, and the timeliness of initiating etiology‐specific therapy, further modulate the probability of recompensation [35, 40, 77, 78].

Several groups have integrated these factors into multivariable prediction models (summarized in Table 2). El‐Sherif et al. previously described the use of the BE3A score to predict recovery from HCV‐related decompensated cirrhosis [61]. A study by Kim et al. in Korea identified six independent predictors associated with recompensation in decompensated HBV‐related cirrhotic patients receiving antiviral therapy and constructed the BC2AID score, which has been proven to have greater stratification and accuracy than traditional prognostic models such as the MELD score and Child‒Pugh score [35]. A prospective study also proposed the Brec‐PAS score for HBV, which provided good prediction for long‐term recompensation [75]. While these tools generally demonstrate that patients with less severe initial decompensation and better preserved liver function are more likely to achieve recompensation, robust, prospective validation in large, independent cohorts is still lacking.

**TABLE 2:** Prediction scores of cirrhosis recompensation.

Prediction score	Etiology and patient	Components	Model performance	Limitations
BE3A score (2018) [61]	HCV‐related decompensated cirrhosis (Child–Pugh Class B or C)	• BMI < 25 kg/m^2^ • No encephalopathy • No ascites • ALT > 1.5 × ULN • ALB > 3.5 g/dL 1 point for each	AUROC 0.75 for predicting improvement to Child–Pugh A within 36 weeks. Score 4‐5: 75% success; Score 0‐1: < 5% success. Outperformed MELD (AUROC 0.56)	1. Short follow‐up period. 2. Lacks external validation. 3. Generalizability limited. 4. Excluded patients with very low platelet count, significant jaundice, or low creatinine clearance. 5. Based on older DAAs regimens
BC2AID score (2022) [35]	HBV‐related decompensated cirrhosis (Child–Pugh score ≥ 7)	• Bilirubin ≤ 5 mg/dL • No complications (refractory ascites or encephalopathy) • AFP ≥ 50 ng/mL • ALT ≥ 200 IU/L • INR ≤ 1.5 • Duration from decompensation to NAs therapy ≤ 6 months 1 point for each except duration (2 points)	AUROC 0.813 for predicting recompensation (Child–Pugh score of 5) within 1 year. Score 6–7: > 90% recompensation rate; Score 0–2: < 10% recompensation rate. Outperformed Child–Pugh, MELD, MELD‐Na, and BE3A scores (all *p* < 0.05)	1. Retrospective design with potential selection bias. 2. Requires exclusion of HCC before applying the model. 3. Needs validation in broader etiologies and diverse cohorts
Brec‐PAS score (2024) [75]	HBV‐related decompensated cirrhosis (with ascites as the initial decompensated event)	Brec‐PAS Score = (0.02 × Platelet count (10^9^/L)) + (0.07 × Albumin (g/L)) + (0.13 × Sodium (μmol/L)) − 22.76	AUROC 0.749 for predicting recompensation (by Baveno VII criteria) by Week 120 of therapy. A score > −0.35 identified a high‐probability group (75.5% recompensation rate vs. 34.6% in the low‐probability group). Outperformed MELD (AUROC 0.629, *p* = 0.002) and FIB‐4 scores	1. Requires on‐treatment data. 2. Generalizability (derived from a cohort where ascites was the initial decompensation event). 3. Selection bias. 4. Retrospective study design with a significant portion of patients lost to extended follow‐up (38.2%)

*Note:* AFP, alpha‐fetoprotein; ALB, albumin; ALT, alanine aminotransferase; HCC, hepatocellular carcinoma; MELD, model for end‐stage liver disease.

Abbreviations: BMI, body mass index; DAAs, direct‐acting antiviral agents; HBV, hepatitis B virus; HCV, hepatitis C virus; INR, International Normalized Ratio; NAs, nucleoside analogs; ULN, upper limit of normal.

## 6. Long‐Term Prognosis and HCC Development in Recompensated Patients

The Baveno VII criteria provide a framework for defining recompensation, yet the long‐term outcomes for these patients remain incompletely characterized, with current evidence presenting inconsistent conclusions.

Evidence supports the notion that recompensation confers a durable clinical benefit. Wang et al. observed that 91.2% of recompensated patients with hepatitis B cirrhosis experienced no further decompensation events at 144 weeks of follow‐up [14]. Similarly, He et al. reported that 71.6% of patients who remained free of a second decompensation for 2 years after initial decompensation maintained a stable recompensated state over 6 years, with a low cumulative 6‐year mortality rate of 12.6% [36]. A large global real‐world cohort study of 4701 HBV‐related cirrhotic patients further substantiated the prognostic value of recompensation. While compensated patients had slightly higher 3‐ and 5‐year transplant‐free survival rates than recompensated patients, this difference was not statistically significant. Crucially, compared to compensated patients, decompensated patients had an approximately sixfold higher risk of mortality or requiring LT [41]. This aligns with findings from Yuan et al. in ACLF patients, which showed that those who reverted to a recompensated cirrhosis had clinical features and a prognosis comparable to patients with compensated cirrhosis [59].

Despite these positive outcomes, recompensation does not equate to a complete recovery, and the risk of disease progression persists. In a study of 199 decompensated patients with hepatitis B cirrhosis in China, 78 experienced recurrent decompensation, and the 2‐, 3‐, 4‐, and 5‐year cumulative re‐decompensation rates were 14.1%, 23.1%, 29.1%, and 34.2%, respectively [79]. In addition, compared to compensated patients, the risk of developing ACLF in recompensated individuals remains significantly greater [31]. This persistent risk may be partly attributable to incomplete regression of the portosystemic collateral circulation established during the decompensated phase, which can lead to the recurrence of portal hypertension‐related complications if underlying risk factors are not controlled [80, 81]. Therefore, while achieving recompensation can lead to favorable clinical outcomes, efforts should still be made to prevent decompensation in patients with early‐stage cirrhosis, and recompensated patients should be treated proactively to prevent recurrent decompensation.

The impact of recompensation on HCC risk is not fully defined, and reported incidence rates vary widely due to differences in population etiology, baseline liver disease severity, duration of follow‐up, and the specific recompensation criteria employed between studies (Table 1). Several studies on hepatitis B cirrhosis indicate a favorable trend. A retrospective study revealed that recompensated individuals with hepatitis B cirrhosis had a significantly decreased risk of HCC compared to those with decompensated cirrhosis, similar to compensated patients (2‐, 4‐, and 6‐year cumulative incidence of HCC: 1.3%, 5.4%, and 20% in the compensated group; 1.2%, 5.2%, and 24.5% in the recompensated group; and 2.1%, 23.6%, and 41.8% in the decompensated group) [43]. Another study by He et al. corroborated that the HCC risk in recompensated patients was lower than in those who did not achieve recompensation [38]. In contrast, Hofer et al. did not observe a significant reduction in HCC risk following recompensation in patients with alcohol‐associated cirrhosis [39]. Kim et al. reported that nearly a quarter of recompensated HBV‐infected patients developed HCC over the long term, which accounted for the majority of deaths in this group [35]. These data underscore that achieving recompensation does not eliminate the risk of HCC. Consequently, lifelong surveillance for HCC remains essential for all recompensated patients.

## 7. Challenges of Cirrhosis Recompensation

### 7.1. Elucidation of the Mechanisms Underlying Recompensation

The pathophysiological processes that enable the transition from decompensated to recompensated cirrhosis remain incompletely understood. While the mechanisms of liver fibrosis regression have been extensively studied, the shift from decompensation to recompensation involves more than just fibrosis resolution, necessitating the reversal of portal hypertension and the resolution of its clinical complications. Research in this area is challenging, as the high risk of liver biopsy in decompensated patients limits the availability of histological data. Consequently, there is a pressing need to develop noninvasive methods to monitor and predict recompensation. Hsu et al. provided insights from animal models. Their findings revealed that upon cessation of injury in cirrhotic rat models, while collateral shunting decreased, the abnormal vascular architecture persisted in a structurally intact but collapsed state, preventing the complete normalization of portal pressure [81]. This finding aligns with clinical observations of residual portal hypertension [46]. Similarly, Olivas et al. demonstrated in a prospective small‐cohort study that persistent esophageal varices and portosystemic shunts may result from structurally residual collateral circulation, though these remnants might be hemodynamically inactive [82].

### 7.2. Clarifying the Definition of Recompensation for Cirrhosis With Different Etiologies

While the Baveno VII consensus provides a standardized framework for defining cirrhosis recompensation, its practical application across all liver diseases remains challenging. The consensus specifically outlines etiological control for hepatitis C, hepatitis B, and alcohol‐associated cirrhosis. A primary controversy is whether the concept applies to diseases without a readily “eliminable” cause. Emerging evidence has demonstrated that sustained clinical improvement observed across various etiologies can significantly improve survival rates, which calls for a broader definition of recompensation criteria that encompasses a wider spectrum of chronic liver diseases. Finally, alongside these definitional expansions, a critical ethical consideration emerges regarding the delisting of recompensated patients from WL. As the underlying pathophysiology may not be fully reversed, these patients consequently remain at risk for disease progression, and a sudden deterioration could jeopardize their timely access to transplantation [83].

### 7.3. Determining Specific Cutoff Values for Liver Function Improvement

The Baveno VII consensus emphasizes the need for steady improvement in liver function (as reflected by ALB, bilirubin, and INR) but does not specify precise biochemical thresholds. A study by Wang et al. proposed that achieving the biochemical range of Child–Pugh A and/or a MELD score < 10 could serve as practical benchmarks. However, as this study was conducted specifically in decompensated patients with HBV‐related cirrhosis where ascites was the initial decompensation event, its generalizability to other etiologies requires validation [14]. Similarly, the stipulated 12‐month complication‐free period lacks robust evidence. It remains unclear whether this 1‐year threshold is universally applicable or requires individualization.

### 7.4. The Challenge of Drug Withdrawal in Defining Recompensation

The Baveno VII requirement for complete withdrawal from diuretics, lactulose, or rifaximin may not align with clinical reality, as many patients achieve long‐term stability on minimal pharmacologic support. This is particularly relevant for nonacute decompensation (NAD), a state often managed with medications where patients still face a 42% risk of progressing to acute decompensation (AD) [84]. However, this study failed to show competing risk analysis, which is crucial for accurately delineating the distinct clinical impacts of AD vs. NAD [85]. This conceptual tension is particularly evident in post‐TIPS patients, where the discontinuation of prophylactic lactulose or rifaximin solely to satisfy recompensation criteria may inadvertently precipitate HE—an outcome more attributable to the TIPS procedure than to true disease progression [45].

Emerging data challenge the necessity of absolute drug cessation. A recent study applied expanded criteria for recompensation in cirrhotic patients with curable etiologies (alcohol, HCV, and HBV), allowing the continued use of lactulose/rifaximin and/or low‐dose diuretics. Compared with the recompensation rate defined traditionally (7%), 37.6% of the patients met the expanded recompensation criteria without compromising survival outcomes [30]. While insightful, it is based on a nonvalidated expansion and utilizes drugs whose beneficial effects are well‐established. This supports prioritizing functional clinical stability over absolute drug withdrawal as the primary endpoint for recompensation. Similarly, the role of nonselective beta‐blockers (NSBBs) in recompensated cirrhosis remains underexplored, as their use often addresses pre‐existing high‐risk varices rather than a sign of incomplete hepatic recovery [84, 86]. These observations collectively prompt a re‐evaluation of the recompensation paradigm: namely, whether the goal should be redefined from a rigid benchmark of “cure” to the pragmatic management of sustained “disease control,” even when maintained with pharmacotherapy.

### 7.5. The Effect of TIPS on Recompensation

While the Baveno VII consensus excluded the recovery status following TIPS from the definition of recompensation, emerging evidence challenges this exclusion by demonstrating TIPS’s potential to facilitate clinical recovery [9]. In patients with adequately controlled underlying etiologies, TIPS may promote recompensation by directly reducing portal hypertension and improving gut microbiota dysbiosis, thereby mitigating systemic inflammation [87]. Among patients who achieve clinical improvement after TIPS, markers of hepatocyte apoptosis/necrosis (M30 and M65) are significantly reduced, correlating with better prognosis [47].

Clinically, Gao et al. reported 31% (20/64) of patients achieved recompensation post‐TIPS, particularly in younger patients achieving a postprocedure portosystemic pressure gradient ≤ 12 mmHg [42]. Similarly, Bai et al. found that 20.3% of 621 TIPS recipients attained recompensation within 12 months, with a significantly better 5‐year survival (42.1% vs. 11.7%, *p* < 0.001) [52]. Prospective data from Sánchez et al. (*n* = 116) reinforced these findings, showing a 24% recompensation rate at 1 year, with subsequent HCC incidence and survival rates comparable to those of compensated patients [45].

However, the interpretation of TIPS‐related recompensation data requires careful consideration of several limitations. First, the net effect of TIPS is often confounded by heterogeneous study populations with varied etiologies and inconsistent reporting on whether these underlying causes were sufficiently controlled. Second, unlike etiological recompensation (e.g., SVR), the hemodynamic improvement from TIPS may be undermined by ongoing progression of liver fibrosis if the primary etiology persists, potentially leading to unstable recompensation. Third, the high incidence of post‐TIPS HE (20%–40% at 1 year) often requires ongoing preventative therapy (e.g., lactulose/rifaximin), which complicates the assessment of recompensation [88]. Therefore, future research should prioritize prospective comparisons between an etiology‐only approach and a combined etiology/TIPS approach, and the recompensation criteria should be refined to take TIPS‐specific challenges into consideration.

### 7.6. Exploring the Impact of Other Therapeutic Approaches on Recompensation and Long‐Term Prognosis of Patients Who Achieve Recompensation

While etiological control is the cornerstone of recompensation, the role of adjuvant therapies—such as endoscopic variceal therapy, statins, nutritional support, and ALB supplementation—in facilitating recompensation remains underexplored and warrants further investigation.

## 8. Conclusion

In conclusion, evidence indicates that a subset of patients with decompensated cirrhosis can achieve clinical recompensation following etiological control, leading to resolved clinical complications, improved liver function, and sustained stability. However, this state is not equivalent to a cure. The likelihood of recompensation is diminished in patients with advanced disease, and even those who meet the current criteria of recompensation still face a risk of future decompensation due to persistent pathophysiological abnormalities. Current strategies are limited by the underdeveloped role of histology in assessment and a lack of robust predictive biomarkers or validated prognostic models; the optimal cutoff values for biochemical improvement need to be further explored. Therefore, the establishment of etiology‐specific recompensation criteria, the development of accurate prediction models for early identification of high‐risk patients, and the implementation of personalized treatment strategies are particularly crucial. Future research is needed to expand this emerging paradigm by elucidating its potential mechanisms, refining prognostic indicators, and characterizing the long‐term outcomes of recompensated patients.

**Nomenclature**
ACLF: acute‐on‐chronic liver failure
AD: acute decompensation
AFP: alpha‐fetoprotein
AIH: autoimmune hepatitis
ALB: albumin
ALD: alcoholic liver disease
ALP: alkaline phosphatase
ALT: alanine aminotransferase
AST: aspartate aminotransferase
BMI: body mass index
DAAs: direct‐acting antiviral agents
EGVB: esophageal and gastric variceal bleeding
HBV: hepatitis B virus
HCC: hepatocellular carcinoma
HCV: hepatitis C virus
HDV: hepatitis D virus
HE: hepatic encephalopathy
HVPG: hepatic venous pressure gradient
IgG: immunoglobulin G
INR: International Normalized Ratio
LDL‐C: low‐density lipoprotein‐cholesterol
LT: liver transplantation
MAP: mean arterial pressure
MASH: metabolic dysfunction–associated steatohepatitis
MASLD: metabolic dysfunction–associated steatotic liver disease
MELD: model for end‐stage liver disease
miRNA: microRNA
NAD: nonacute decompensation
NAs: nucleos(t)ide analogs
NLR: neutrophil‐to‐lymphocyte ratio
NSBBs: nonselective beta‐blockers
PBC: primary biliary cholangitis
PLT: platelet
SVR: sustained virologic response
TIPS: transjugular intrahepatic portosystemic shunt
UDCA: ursodeoxycholic acid
ULN: upper limit of normal
WL: waiting list

## Author Contributions

Dan Zhou, Kunyi Ba, and Tingting Jia: writing–original draft and visualization. Yi Shen and Li Yang: reviewing and editing.

## Funding

This study was funded by the 135 Project for Disciplines of Excellence, West China Hospital, Sichuan University (No. ZYGD23031 to Li Yang), and the Postdoctoral Research Fund of West China Hospital, Sichuan University (No. 2024HXBH120 to Yi Shen).

## Disclosure

All authors reviewed the manuscript and approved the submission.

## Ethics Statement

The authors have nothing to report.

## Conflicts of Interest

The authors declare no conflicts of interest.

## Data Availability

Data sharing is not applicable to this article as no datasets were generated or analyzed during the current study.
